# Altered Neural Network Connectivity Predicts Depression in *de novo* Parkinson’s Disease

**DOI:** 10.3389/fnins.2022.828651

**Published:** 2022-03-04

**Authors:** Jianxia Xu, Yubing Chen, Hui Wang, Yuqian Li, Lanting Li, Jingru Ren, Yu Sun, Weiguo Liu

**Affiliations:** ^1^Department of Neurology, The Affiliated Brain Hospital of Nanjing Medical University, Nanjing, China; ^2^Department of Neurology, Lianyungang Hospital of Traditional Chinese Medicine, Lianyungang, China; ^3^Department of Neurology, Affiliated BenQ Hospital of Nanjing Medical University, Nanjing, China; ^4^International Laboratory for Children’s Medical Imaging Research, School of Biological Sciences and Medical Engineering, Southeast University, Nanjing, China

**Keywords:** Parkinson’s disease, depression, neural network, independent component analysis, drug-naïve

## Abstract

**Background:**

Depression, one of the most frequent non-motor symptoms in Parkinson’s disease (PD), was proposed to be related to neural network dysfunction in advanced PD patients. However, the underlying mechanisms in the early stage remain unclear. The study was aimed to explore the alterations of large-scale neural networks in *de novo* PD patients with depression.

**Methods:**

We performed independent component analysis (ICA) on the data of resting-state functional magnetic resonance imaging from 21 *de novo* PD patients with depression (dPD), 34 *de novo* PD patients without depression (ndPD), and 43 healthy controls (HCs) to extract functional networks. Intranetwork and internetwork connectivity was calculated for comparison between groups, correlation analysis, and predicting the occurrence of depression in PD.

**Results:**

We observed an ordered decrease of connectivity among groups within the ventral attention network (VAN) (dPD < ndPD < HCs), mainly located in the left middle temporal cortex. Besides, dPD patients exhibited hypoconnectivity between the auditory network (AUD) and default mode network (DMN) or VAN compared to ndPD patients or healthy controls. Correlation analysis revealed that depression severity was negatively correlated with connectivity value within VAN and positively correlated with the connectivity value of AUD-VAN in dPD patients, respectively. Further analysis showed that the area under the curve (AUC) for dPD prediction was 0.863 when combining the intranetwork connectivity in VAN and internetwork connectivity in AUD-DMN and AUD-VAN.

**Conclusion:**

Our results demonstrated that early dPD may be associated with abnormality of attention bias and especially auditory attention processing. Altered neural network connectivity is expected to be a potential neuroimaging biomarker to predict depression in PD.

## Introduction

Parkinson’s disease (PD) affects 2–3% of the population over 65 years of age, which is the second most common neurodegenerative disorder next to Alzheimer’s disease (Poewe et al., 2017). As one of the most frequent non-motor symptoms, the overall prevalence of depression in PD (dPD) patients can reach up to 17–35% (Reijnders et al., 2008; Aarsland et al., 2011). As for the etiology and pathogenesis of depression in PD, one of the most recognized is the neurotransmitter theory. Depressive symptoms in PD are thought to be associated with decreased neurotransmission in the basal ganglia (dopamine) and locus coeruleus (dopamine and norepinephrine) (Ballanger et al., 2012; Skidmore et al., 2013). However, how neurotransmitters or other risk factors contribute to the onset and progression of dPD remains largely ambiguous. A better understanding of the underlying pathogenetic mechanisms is crucial for the early diagnosis and treatment considering the serious impact on patients’ quality of life.

Resting-state functional magnetic resonance imaging (rsfMRI) is a non-invasive technique, which can be used to detect how aberrant brain activity or functional connectivity between core areas leads to clinical depressive symptoms in PD. Previous studies using the amplitude of low-frequency fluctuation (ALFF) and regional homogeneity (ReHo) methods showed that dPD was related to abnormal neural activity in several specific brain regions, including the supplementary motor area (SMA) and the prefrontal-limbic network (Wen et al., 2013; Luo et al., 2014; Wang et al., 2020). Seed-based functional connectivity (FC) analysis found that dPD patients showed decreased FC in the insula network (Huang et al., 2020) and increased FC between the left median cingulated cortex and the default mode network (DMN) in dPD patients (Hu et al., 2015). In recent years, researchers have become increasingly interested in the role of abnormal connectivity among large-scale functional neural networks in the pathophysiology of neurodegenerative diseases, instead of within a single, discrete brain region. Hence, independent component analysis (ICA) was introduced to explore alterations of network connectivity in dPD.

Independent component analysis is a data-driven whole-brain blind source separation method that can separate a multivariate signal into independent networks, thus avoiding the inherent deviation in selecting specific seeds (Smith et al., 2009). Our previous study demonstrated that dPD patients exhibited altered intra- and internetwork connectivity, with the involvement of the basal ganglia network, default mode network (DMN), left frontoparietal network (LFPN), and salience network (SN; Wei et al., 2017). More recently, a more systematic study with a larger sample size confirmed that dysregulation of the triple network may facilitate the development of depression in PD (Liao et al., 2020). However, the patients in the previous studies were not newly diagnosed and had taken anti-parkinsonism medications before enrollment. Dopaminergic therapy has been proved to result in reorganization of functional integration of the DMN and sensorimotor pathways in PD patients (Zhong et al., 2019; Goelman et al., 2021). Moreover, the dosage of anti-PD medication is associated with depression severity in PD patients (Lee et al., 2018). Thus, neural network studies on drug-naïve PD patients would be necessary to clarify the neuro-pathophysiological mechanisms of depressive symptoms in PD.

In this study, we applied the ICA approach to rsfMRI data to investigate intrinsic connectivity within and between several classical functional networks, focusing on the drug-naïve PD patients with depression. We hypothesize that disrupted network function may contribute to depression in PD patients.

## Materials and Methods

### Participants

Parkinson’s disease patients recruited from the Affiliated Brain Hospital of Nanjing Medical University were diagnosed by an experienced neurologist according to the United Kingdom Parkinson’s Disease Society Brain Bank diagnostic criteria (Hughes et al., 1992). The exclusion criteria were: (1) history of taking anti-parkinsonism or antidepressant drugs; (2) history of cerebrovascular disorders, head injury, seizure, hydrocephalus, intracranial mass, previous neurological surgery, or other neurologic diseases; (3) dementia; (4) history of psychiatric diseases except depression; or (5) poor MRI image quality or excessive head motion. All the patients were followed up for at least 1 year to confirm the diagnosis according to the response to the dopaminergic therapy and disease evolution. Healthy controls (HCs) with no history of neurologic or psychiatric diseases were recruited and matched to PD patients for age, gender, and education. Fifty-five *de novo* PD patients and forty-three HCs were finally included in this study. All the participants provided written informed consent, completed all the clinical assessments and MRI scans during the baseline visit. The study was approved by the Medical Ethics Committee of the Affiliated Brain Hospital of Nanjing Medical University.

### Clinical Assessments

Depression in PD was diagnosed using the Diagnostic and Statistical Manual of Mental Disorders, Fifth Edition (DSM-V) criteria by an experienced psychiatrist. The severity of depression was quantified with the 17-item Hamilton Depression Rating Scale (HAMD-17). dPD patients were required to have a HAMD-17 score higher than 14 (Leentjens et al., 2000). A total of 21 patients were finally diagnosed with dPD. In addition, disease severity was evaluated by Hoehn and Yahr (H&Y) staging scale and the motor section of the Unified Parkinson’s Disease Rating Scale (UPDRS-III). Cognition was assessed using the Mini-Mental State Examination (MMSE), and MMSE scores ≥24 were required for each participant. All the assessments were conducted immediately before the MRI scan.

### Magnetic Resonance Imaging Acquisition

Magnetic resonance imaging was performed using a 3T MRI scanner (Siemens, Verio, Munich, Germany). All participants lay supine with their head fixed by foam pads with a standard birdcage head coil to minimize head movement. The participants were instructed to remain as still as possible and to close their eyes while remaining awake without thinking of anything. Axial anatomical images were acquired using a T1 fluid-attenuated inversion recovery sequence with the following parameters: repetition time (TR) = 2,530 ms; echo time (TE) = 3.34 ms; field of view (FOV) = 256 mm × 256 mm; matrix = 256 × 192; slice thickness/gap = 1.33/0.5 mm; flip angle (FA) = 7 degrees; bandwidth = 180 HZ/PX; 128 slices covered the whole brain, for image registration and functional localization. Functional images were subsequently collected in the same slice orientation with a gradient-recalled echo-planar imaging pulse sequence. A total of 240 volumes were obtained (TR = 2,000 ms; TE = 30 ms; FOV = 220 mm × 220 mm; matrix = 64 × 64; thickness/gap = 3.5/0.63 mm; FA = 90 degrees; bandwidth = 2,232 HZ/PX; slice numbers = 31).

### Data Preprocessing

Preprocessing of the fMRI data was carried out using SPM12^1^ and DPARSF.^2^ The first 10 volumes of each functional section were discarded for magnetization equilibrium. The remaining 230 volumes were then corrected for slice timing using the middle slice as a reference, realigned for head motion correction, segmented into white matter, gray matter, and cerebrospinal fluid (CSF) using tissue probability maps derived from the T1 images, normalized into the standard Montreal Neurological Institute space using diffeomorphic anatomical registration through exponentiated Lie algebra (DARTEL), resampled to a 3 mm × 3 mm × 3 mm voxel size, and smoothed with a 6 mm full-width at half-maximum (FWHM) Gaussian kernel (Zhou C. et al., 2020). To further minimize the potential effects of head motion, we excluded participants with a mean framewise displacement (FD) > 0.5 mm or whose head motion exceeded a maximum translation of 3 mm or rotation of 3° from the further analysis, referring to the previous study (Navalpotro-Gomez et al., 2020), resulting in the exclusion of one dPD patient, three ndPD patients, and two HCs. No significant differences in head motion were found between dPD, ndPD, and HCs groups (ANOVA, *P* = 0.12).

### Independent Component Analysis

The preprocessed resting-state data of all subjects were decomposed into functional networks by applying spatial group independent component analysis (ICA) implemented in the Group ICA of the fMRI Toolbox (GIFT v4.0b).^3^ The procedures included three steps: (1) data reduction, (2) group ICA, and (3) back-reconstruction. In this study, 32 ICs were estimated through the minimum description length (MDL) criteria (Jafri et al., 2008). The dimensionality of the datasets was reduced using principal component analysis (PCA). Second, the Infomax algorithm was used to run the ICA, and repeated 20 times in ICASSO to ensure stability and validity (Bell and Sejnowski, 1995; Himberg et al., 2004). Finally, subject-specific spatial maps and time courses were generated using the GICA back-reconstruction approach (Calhoun et al., 2001). Each back-reconstructed component consists of a spatial z-map reflecting the functional connectivity pattern across space and an associated time course reflecting component’s activity across time. The resulting 32 ICs were sorted with each of the RSN templates by spatial correlation, and the component with the highest correlation coefficient was selected and reconfirmed by visual inspection (Wei et al., 2017). Nine components extracted from all participants were finally identified as functional networks: auditory network (AUD), default mode network (DMN), dorsal attention network (DAN), left frontoparietal network (LFPN), right frontoparietal network (RFPN), sensorimotor network (SMN), ventral attention network (VAN), visual network (VIS), and salience network (SN).

### Intranetwork Connectivity Analysis

**FIGURE 1:**
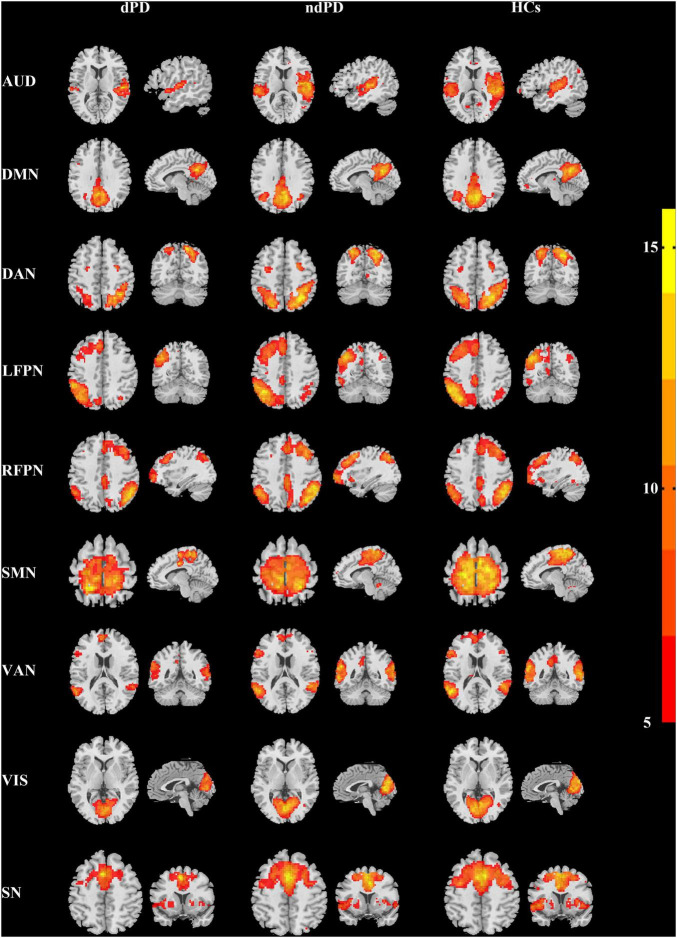
Spatial maps of the identified functional networks (one-sample *t*-test, *P* < 0.001, FDR-corrected). AUD, auditory network; DMN, default mode network; DAN, dorsal attention network; LFPN, left frontoparietal network; RFPN, right frontoparietal network; SMN, sensorimotor network; VAN, ventral attention network; VIS, visual network; SN, salience network.

Intranetwork connectivity within each functional network was calculated using the reconstructed spatial z-maps. Voxel-wise one-sample *t*-tests were performed on the spatial z-maps across all participants for each component in SPM12 to determine regions that are positively significantly integrated into each network [*P* < 0.001, false discovery rate (FDR)-corrected] (Wei et al., 2017; Figure 1). One-way analysis of variance (ANOVA) was used to calculate the comparisons among dPD, ndPD, and HCs groups, followed by *post-hoc* two-sample *t*-tests. Multiple comparisons were performed by using FDR correction.

### Internetwork Connectivity Analysis

In order to evaluate the internetwork connectivity, the subject-specific time courses of the selected ICs were postprocessed by detrending, despiking, filtering with a high cutoff frequency of 0.15 HZ, and regressing out the head motion parameters (Liao et al., 2020). Pair-wise Pearson’s correlations between the selected ICs were computed and converted to *z*-scores *via* Fisher’s z-transformation. We extracted the transformed z-scores of each network pair for the three groups and performed ANOVA tests (*P* < 0.05, FDR-corrected), followed by *post-hoc* two-sample *t*-tests to compare the internetwork connectivity between groups (*P* < 0.05, Bonferroni-corrected).

### Statistical Analysis

Statistical analyses were calculated using SPSS Statistic 24.0 (Chicago, IL, United States). Two-sample *t*-test or Mann-Whitney U test was applied to compare two groups. One-way ANOVA or Kruskal–Wallis test was performed to compare HCs, ndPD, and dPD groups. Chi-squared test was used to compare categorical variables such as gender. Pearson correlation analyses were conducted to assess the relationships between the altered intra- or internetwork connectivity and HAMD-17 scores. Simple and multiple binary logistic regression was used to determine the risk factors associated with depression in PD patients. The predictive value of individual or combined brain network indices for depression in PD was assessed by the receiver operating characteristic (ROC) curve, and the area under the curve (AUC) was calculated simultaneously. The threshold for statistical significance was set at *P* < 0.05.

## Results

### Demographic and Clinical Characteristics

A total of 34 ndPD patients, 21 dPD patients, and 43 healthy controls were eventually included in the further analysis. There were no significant differences in age, gender, education, MMSE scores, and mean FD among the three groups. No significant differences in disease duration, H&Y stage, and UPDRS-III scores were observed between dPD and ndPD groups. In contrast, the HAMD-17 scores of dPD patients were significantly higher than that of ndPD patients or healthy controls (Table 1).

**TABLE 1:** Demographic and clinical information of the participants.

	dPD *n* = 21	ndPD *n* = 34	HCs *n* = 43	*P*-value
Age (years)	59.33 ± 5.73	59.79 ± 8.40	59.44 ± 5.86	0.963^a^
Gender (male/female)	9/12	14/20	15/28	0.780^b^
Education (years)	9.05 ± 2.94	9.56 ± 2.67	10.51 ± 3.30	0.150^a^
Disease duration (years)	2.05 ± 1.40	1.97 ± 1.13	–	0.823^c^
H & Y	2.00(1.50, 2.00)	1.50(1.50, 2.00)	–	0.199^d^
UPDRS-III	28.57 ± 8.97	24.56 ± 8.28	–	0.097^c^
MMSE	27(25.5, 29)	29(28, 29)	29(27, 30)	0.056^e^
HAMD-17	17.43 ± 4.87	3.68 ± 1.75	1.21 ± 1.64	0.000^a,^ *

*dPD, Parkinson’s disease with depression; ndPD, Parkinson’s disease without depression; HCs, healthy controls; H & Y stage: Hoehn and Yahr stages; UPDRS-III, the motor section of the Unified Parkinson’s Disease Rating Scale; MMSE, Mini-Mental State Examination; and HAMD-17, 17-item Hamilton Depression Rating Scale.*

Parametric variables are presented as mean ± SD, and non-parametric variables are presented as median (interquartile range). *P < 0.05.

*^a^One-way ANOVA.*

*^b^Chi-squared test.*

*^c^Two-sample t-test.*

*^d^Mann–Whitney U test.*

*^e^Kruskal–Wallis test.*

### Intranetwork Connectivity Analysis

**FIGURE 2:**
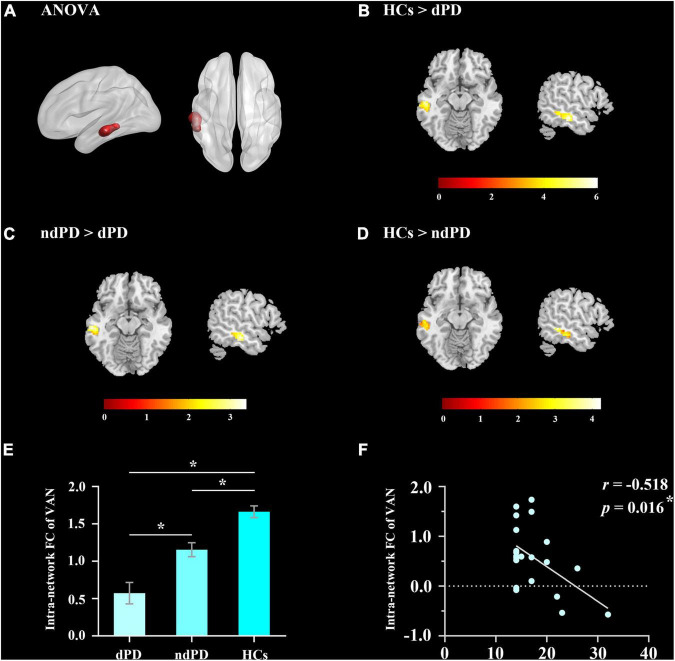
Results of intranetwork analysis. **(A)** The ANOVA result for altered intranetwork connectivity among dPD, ndPD, and HCs groups (*P* < 0.05, FDR-corrected). **(B,C)** dPD group showed significantly decreased connectivity in the left middle temporal cortex of VAN compared to ndPD and HCs group (*P* < 0.05, FDR-corrected). **(D)** ndPD group showed significantly decreased connectivity in this brain region compared to HCs group (*P* < 0.05, FDR-corrected). **(E)** FC value histogram for the intranetwork connectivity in VAN. Asterisks indicate a significant group difference (*post-hoc t*-tests, *P* < 0.05, FDR-corrected). **(F)** Correlation between the FC value of intranetwork connectivity in VAN and HAMD-17 scores in dPD patients. FC, functional connectivity; VAN, ventral attention network.

Analysis of variance results showed that the VAN exhibited a significant difference in intranetwork connectivity among dPD, ndPD, and HCs groups (*P* < 0.05, FDR-corrected) (Figure 2A). *Post-hoc t*-tests indicated that dPD group showed significantly decreased connectivity in the left middle temporal cortex of the VAN compared to ndPD and HCs group (*P* < 0.05, FDR-corrected). Besides, ndPD group also showed significantly decreased connectivity in this brain region compared to HCs group (*P* < 0.05, FDR-corrected) (Figures 2B–E and Table 2). No significant differences were found within other functional networks.

**TABLE 2:** *Post-hoc* comparisons of intranetwork connectivity among PD patients with depression (dPD), Parkinson’s disease without depression (ndPD), and healthy controls (HCs) groups.

Brain region (AAL)	BA	Cluster size (voxels)	*T*-value	MNI coordinate		
				*x*	*y*	*z*
**HCs > dPD**						
Temporal_Mid_L	–	90	6.02	−57	−24	−15
**HCs > ndPD**						
Temporal_Mid_L	–	70	4.21	−54	−42	−6
**ndPD > HCs**						
Temporal_Mid_L	21	58	3.37	−60	−24	−12

*BA, Brodmann area; MNI, Montreal Neurological Institute; Temporal_Mid_L, left middle temporal cortex.*

### Internetwork Connectivity Analysis

**FIGURE 3:**
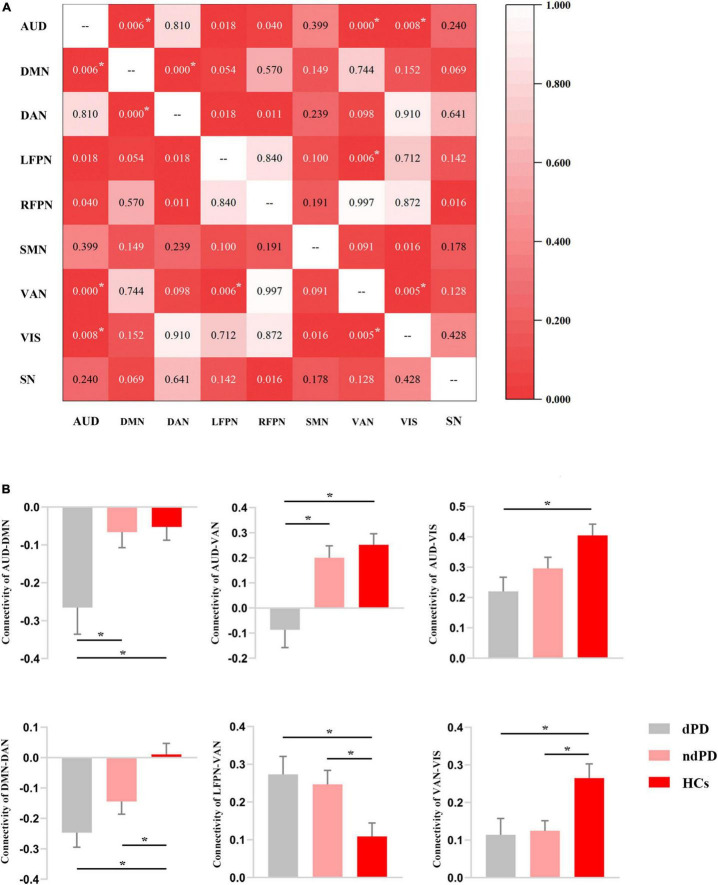
Results of internetwork analysis. **(A)** The matrix for the ANOVA results among dPD, ndPD, and HCs groups in terms of internetwork connectivity. The figure in the matrix represents the *P*-value of ANOVA. Asterisks indicate a significant difference (*P* < 0.05, FDR-corrected). **(B)** FC value histogram for the internetwork connectivity and *post-hoc* comparisons based on the significant ANOVA results. Asterisks indicate a significant difference (*P* < 0.05, Bonferroni-corrected).

Analysis of variance results showed that connectivity between AUD and DMN/VAN/VIS, between DAN and DMN, and between VAN and LFPN/VIS differed significantly among the three groups (*P* < 0.05, FDR-corrected) (Figure 3A). *Post-hoc t*-tests showed that dPD group compared to ndPD group had decreased connectivity between AUD and DMN/VAN (*P* < 0.05, Bonferroni-corrected). Compared to HCs group, dPD group had increased connectivity between LFPN and VAN and decreased connectivity between AUD and DMN/VAN/VIS, between DAN and DMN, and between VAN and VIS (*P* < 0.05, Bonferroni-corrected). Compared to HCs group, ndPD group had increased connectivity between LFPN and VAN and decreased connectivity between DMN and DAN and between VAN and VIS (*P* < 0.05, Bonferroni-corrected) (Figure 3B).

### Correlation Analysis

**FIGURE 4:**
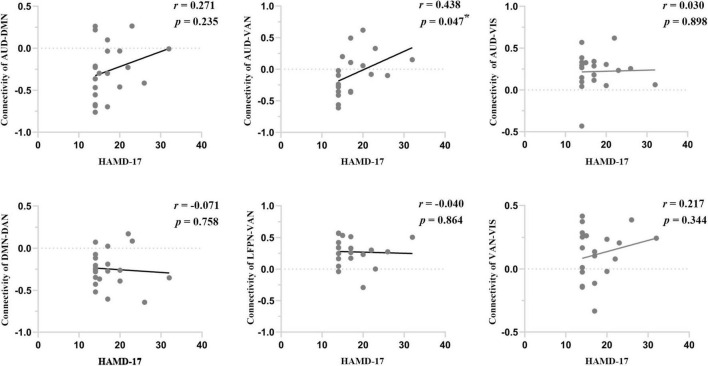
The correlations between internetwork connectivity and HAMD-17 scores in dPD patients. Asterisks indicate a significant correlation (*P* < 0.05).

We performed Pearson correlation analyses between the significantly altered connectivity and HAMD-17 scores. The results showed that intranetwork connectivity in the left middle temporal cortex of the VAN was negatively correlated with HAMD-17 scores in dPD patients (*P* = 0.016, *r* = −0.518) (Figure 2F). In addition, connectivity between AUD and VAN was positively correlated with HAMD-17 scores in dPD patients (*P* = 0.047, *r* = 0.438) (Figure 4).

### Binary Logistic Regression Analysis

Based on the results of the initial comparisons between ndPD and dPD groups in terms of the brain network indices, we included intranetwork connectivity in VAN, internetwork connectivity between AUD and DMN, and between AUD and VAN in the simple binary logistic regression analysis. The results showed that these network indices were significant predictors associated with depression in PD. Multiple binary logistic regression with step-wise selection showed that intranetwork connectivity in VAN and internetwork connectivity between AUD and DMN were independently associated with depression in PD (Table 3).

**TABLE 3:** Simple and multiple binary logistic regression models for the factors associated with depression in Parkinson’s disease (PD).

Variables	Simple			Multiple		
	OR	95% CI	*P*-value	OR	95% CI	*P*-value
Intranetwork FC of VAN	0.188	0.062–0.573	0.003	0.085	0.020–0.364	0.001
Internetwork FC of AUD-DMN	0.065	0.007–0.620	0.018	0.011	0.001–0.193	0.002
Internetwork FC of AUD-VAN	0.039	0.004–0.337	0.003			

*OR, odds ratio; CI, confidence interval; FC, functional connectivity; VAN, ventral attention network; AUD, auditory network; DMN, default mode network; and FC, functional connectivity.*

### Receiver Operating Characteristic Curve Analysis for Diagnosis of Parkinson’s Disease Patients With Depression

**FIGURE 5:**
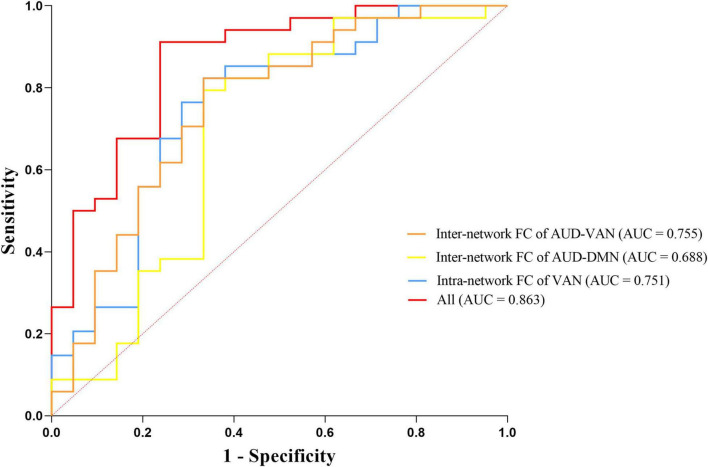
Receiver operating characteristic (ROC) curves for predicting dPD patients by three neuroimaging biomarkers including intranetwork connectivity in VAN, internetwork connectivity between AUD and DMN, and between AUD and VAN.

We selected intranetwork connectivity in VAN, internetwork connectivity between AUD and DMN, and between AUD and VAN as biomarkers to distinguish dPD patients from ndPD patients based on the results of correlation analysis and regression analysis. We found that the area under the curve (AUC) for dPD prediction was 0.751 for intranetwork connectivity in VAN (95% CI = 0.611–0.891, *P* = 0.002), 0.688 for internetwork connectivity between AUD and DMN (95% CI = 0.527–0.848, *P* = 0.020), and 0.755 for internetwork connectivity between AUD and VAN (95% CI = 0.617–0.893, *P* = 0.002), respectively. The accuracy improved (AUC = 0.863) when combining the three brain network indices (95% CI = 0.760–0.965, *P* < 0.001) (Figure 5).

## Discussion

The present study investigated altered connectivity within and between large-scale brain networks in drug-naïve PD patients with depression, thereby excluding the confounding effects of anti-parkinsonism therapy and chronic duration. We observed an ordered decrease of intranetwork connectivity among groups in VAN (dPD < ndPD < HCs), mainly located in the left middle temporal cortex, and the connectivity value was negatively correlated with depression severity in dPD patients. Besides, the internetwork connectivity between AUD and DMN/VAN differed significantly between dPD patients and ndPD patients or healthy controls. Further analysis revealed a positive correlation between the connectivity value of AUD-VAN and depression severity in dPD patients. Importantly, we found the combination of intra- and internetwork connectivity could predict the occurrence of depression in PD with a relatively high diagnostic accuracy.

Ventral attention network is related to stimulus-driven attention orientation and is activated in detecting unexpected stimuli and triggering shifts of attention (Corbetta and Shulman, 2002; Vossel et al., 2014). Specifically, VAN is involved in top-down controlled attentional selection and bottom-up attentional processing through dynamic interaction with DAN (Geng and Vossel, 2013; Vossel et al., 2014), thus mediating externally oriented attention, cognitive and emotional regulation (Kaiser et al., 2015; Mao et al., 2020; Stumme et al., 2020). Previous studies had proved that abnormal function of VAN was implicated in several psychiatric disorders such as major depressive disorder and adolescent depression (Liu et al., 2019; Ayyash et al., 2021). The activation of VAN was triggered in response to violation of expectation, thereby reorienting to environmental stimuli, driving learning, reward, or affective mechanisms (Corbetta et al., 2008). The downregulating of VAN activation resulted in the disruption of reward circuit and in turn the onset of depression. Additionally, depressed patients tend to struggle when disengaging themselves from negative information in the external environment (Gotlib et al., 2004; Kellough et al., 2008). The dysfunction of VAN further results in the negative attention bias and failure to switch the attention to positive incidents for patients (Eizenman et al., 2003), thus aggravating depression. Besides, VAN is anti-correlated with DMN in resting-state brain connectivity (Fox et al., 2005). The hypoconnectivity within VAN may cause an overactive DMN and promote the self-referential processes and negative rumination, which is associated with mood disturbances (Belzung et al., 2015). Moreover, the locus coeruleus/norepinephrine system (LC/NE) system is a monoaminergic neuromodulatory system (Schwarz and Luo, 2015), involved in the release of tonic signals in the brain and is sensitive to reward information (Aston-Jones and Cohen, 2005). A previous study proposed that the signals of the LC/NE paralleled the activity of VAN, and the deactivation of VAN during focused attention may be due to the reduced tonic signals of the LC/NE system (Corbetta et al., 2008). This study combined with our results may further confirm the theory of LC/NE neurotransmitter in the pathophysiological mechanism of depression in PD. Furthermore, our results found the altered connectivity within the VAN was mainly located in the left middle temporal cortex and observed a negative correlation between the connectivity values and depression severity in dPD patients. The middle temporal cortex is a crucial network hub for effective information transmission in the brain, in which the node topological properties and cortical thickness were correlated with depressive symptoms in different stages of PD (Hanganu et al., 2017; Qiu et al., 2021). We speculated that the early targets of the neuropathological damage in dPD tend to be highly interactive brain regions, worsening progressively with the course of the disease.

Dopamine modulated auditory processing in different stages of auditory system from peripheral to central hearing-related regions (Jafari et al., 2020). When dopamine is reduced in the brain, which is the most important pathology in PD, the response of the AUD to sound stimuli would change, thus affecting the appropriate extraction, differentiation, and processing of auditory information (Hoyt et al., 2019). The integrated auditory processing ability supports the normal communication with the environment and helps to balance the emotional reaction. Conversely, the disruption of AUD may lead to emotional disturbances, such as depression. Furthermore, AUD was closely related with other emotion-related networks. Auditory signals are perceived and processed in the auditory cortex for appraisal and identification, then sent to higher-order cortices for the production of a specific affective state in response to the stimulus and the regulation of the affective state and emotional behavior (Zatorre et al., 2002; Phillips et al., 2003). A previous study on patients with major depression found reduced activation in the auditory cortex and frontoparietal attention network during auditory mismatch processing (Zweerings et al., 2019). VAN is involved in the involuntary/exogenous stimulus-driven auditory orienting as previously mentioned (Rossi et al., 2014). The disconnection between AUD and VAN may disrupt bottom-up auditory attention shifts, resulting in the diminishment of detection and orientation toward positive environmental cues for depressed individuals. DMN was thought to be associated with self-referential judgments and to take part in self-regulation of emotional responses through self-focused reappraisal and diversion of attention from emotional arousal stimuli (Ochsner et al., 2004; Eippert et al., 2007). The precuneus had been previously suggested to participate in top-down control of auditory attention (Shomstein and Yantis, 2006), while the ventral medial prefrontal cortex, as one of the subsystems of DMN(Zhou H. X. et al., 2020), is likely to be involved in evaluating the significance of auditory stimuli cues and selecting the appropriate emotional response (Rossi et al., 2014). We speculated that decreased connection between AUD and DMN led to a bias for self-referential thinking at the expense of auditory attention to external stimuli, thus failing to distract the individual’s depressed mood. While correlation analysis showed in dPD patients that connectivity between AUD and VAN increased as depressive symptoms worsened, which indicates a possible functional compensation for the damaged auditory network in early drug-naïve PD patients with depression. Altogether, the results of the present study may suggest early impairment at the level of auditory information processing and auditory attention shifts may be a potential mechanism in depressed PD patients.

Additionally, we also found that both subgroups of PD patients had increased connectivity between LFPN and VAN and decreased connectivity between DAN and DMN and between VAN and VIS compared to HCs. Enhanced connectivity between FPN and VAN was demonstrated to help to capture environmental information in time, so as to realize the flexible cognitive control (Li et al., 2021). The strength of the anti-correlation between DAN and DMN is related to the efficiency of attention regulation (Kelly et al., 2008). The findings in our study indicated a compensatory mechanism for cognition and attention deficits in early PD patients. Furthermore, the decreased connectivity between VIS and VAN may be due to the disruption of the bottom-up triggered visual attention system according to the model of Corbetta and Shulman (2002).

The ROC analysis showed that altered intranetwork connectivity in VAN and internetwork connectivity between AUD and DMN/VAN could be effective biomarkers to differentiate dPD patients from ndPD patients. Despite the higher accuracy of our results to predict dPD patients than the previous studies (Zhu et al., 2016; Li et al., 2017), single imaging marker of rsfMRI might be restricted. Considering that the occurrence and progress of depression in PD may be attributed to many different aspects including cortical atrophy (Hanganu et al., 2017), white matter integrity (Huang et al., 2014) and even genetic heterogeneity (Lin et al., 2017), a combination of different biomarkers may contribute to a more practical and accurate clinical diagnosis of dPD.

Nevertheless, our study has several limitations. First, the relatively small sample size limited the generalizability of our results, for the patients in our study were newly diagnosed and were followed up for at least 1 year to ensure the diagnosis. A larger sample size would be implemented in the future to validate the results. Second, cognition was proposed to be associated with depression, but MMSE was not effective enough to identify the possible cognitive impairment. Thus, more comprehensive neuropsychological tests should be conducted to exclude the confounding effects of cognition. Third, as mentioned above, only using neural network connectivity as a biomarker is restricted, so multimodality imaging, genetic or plasma markers are expected to be involved in our patient cohort.

## Conclusion

In summary, we observed decreased intranetwork connectivity in VAN and internetwork connectivity between AUD and DMN/VAN in *de novo* PD patients with depression, suggesting that the neural network mechanism of dPD may be associated with abnormality of attention bias, especially auditory attention processing. These findings could provide new insights into the potential diagnostic markers and therapeutic targets of depression in PD.

## Data Availability Statement

The original contributions presented in the study are included in the article, further inquiries can be directed to the corresponding author.

## Ethics Statement

The studies involving human participants were reviewed and approved by the Medical Research Ethics Committee of the Affiliated Brain Hospital of Nanjing Medical University. The patients/participants provided their written informed consent to participate in this study.

## Author Contributions

WL designed and organized the research. JX, YC, YL, LL, and JR collected the imaging and assessment scale data. JX, YC, and HW were responsible for formal analysis. JX drafted the manuscript. WL and YS made important revisions to the manuscript. All authors contributed to the article and approved the submitted version.

## Conflict of Interest

The authors declare that the research was conducted in the absence of any commercial or financial relationships that could be construed as a potential conflict of interest.

## Publisher’s Note

All claims expressed in this article are solely those of the authors and do not necessarily represent those of their affiliated organizations, or those of the publisher, the editors and the reviewers. Any product that may be evaluated in this article, or claim that may be made by its manufacturer, is not guaranteed or endorsed by the publisher.

## Footnotes

1
http://www.fil.ion.ucl.ac.uk/spm

2
http://rfmri.org/DPARSF

3
http://icatb.sourceforge.net
